# Scheduling Emergency Physicians Based on a Multiobjective Programming Approach: A Case Study of West China Hospital of Sichuan University

**DOI:** 10.1155/2019/5647078

**Published:** 2019-08-26

**Authors:** Mingying Tan, Junwei Gan, Qunrong Ren

**Affiliations:** ^1^West China Hospital of Sichuan University, Chengdu 610041, China; ^2^School of Economics and Management, Sichuan Tourism University, Chengdu 610100, China; ^3^Business School, Sichuan University, Chengdu 610065, China

## Abstract

In China, emergency room residents (EMRs) generally face high working intensity. It is particularly important to arrange the working shifts of EMRs in a scientific way to balance their work and rest time. However, in existing studies, most of the scheduling models are based on the individual doctor or nurse as a unit, less considering the actuality of operation and management of emergency department (ED) in large public hospitals in China. Besides, the depiction of the hard and soft constraints of EMR scheduling in China is insufficient. So in order to obtain the scientific and reasonable scheduling shifts, this paper considers various management rules in a hospital, physicians' personal preferences, and the time requirements of their personal learning and living and takes the minimum deviation variables from the soft constraints as the objective function to construct a mixed integer programming model with the doctor group as the scheduling unit. The analytic hierarchy process (AHP) is used to determine the weights of deviation variables. Then, IBM ILOG CPLEX 12.8 is used to solve the model. The feasibility and effectiveness of the scheduling method are verified by the actual case from West China Hospital of Sichuan University. The scheduling results can meet the EMRs' flexible work plans and the preferences of the doctor teams for the shifts and rest days. Compared with the current manual scheduling, the proposed method can greatly improve the efficiency and rationality of shift scheduling. In addition, the proposed scheduling method also provides a reference for EMR scheduling in other China's high-grade large public hospitals.

## 1. Introduction

The emergency department (ED) is not only a rescue department for severely ill patients but also a window for a hospital. The medical technology level and service quality of the ED are important aspects of social evaluation of hospitals. As the key department to provide all kinds of critical life support for patients, the ED must be open for 24 hours a day, 365 days a year. So the emergency room residents (EMRs) must work more shifts, often working at night and on weekends. In China, the day shift or the night shift of emergency room doctors is for up to 12 hours. Heavy work intensity and long shift time result in EMRs prone to fatigue, anger, pain, resentment, and other bad emotions [1, 2]. A study shows that 24.5% of emergency physicians are not satisfied with their current work [3]. In China, in recent years, because of the influence of patients' health-seeking habits and rapid growth of patient visits (reaching as much as 810 million in 2017), EMRs in some advanced large public hospitals with good technical equipment condition are more stressed in face of a large flow of emergency patients everyday, such as West China Hospital of Sichuan University (WCH). WCH is one of the largest single-site hospitals in the world and also a leading medical center in China. In the ED of WCH, patient attendance hits 250 thousand, and over 60 thousand patients are rescued from certain death every year. In addition, as a research-oriented hospital, the EMRs of WCH take on the tasks of scientific research, teaching, and instructing students. Therefore, the ED in the most of the large public hospitals in the metropolis of China, represented by WCH, is often one of the most stressful, challenging, and high turnover departments in such hospitals [1, 4]. Therefore, in order to reduce the work pressure of EMRs and balance the work and rest time, it is particularly important to arrange the working time of emergency physicians in a scientific way. The effective arrangements for EMRs are helpful to improve the quality of medical service, optimize the management mechanism of the hospital, and alleviate the conflicts between doctors and patients [5].

The scheduling problem of medical staff is a combinatorial optimization problem. The current research mainly focuses on general medical departments, especially the nurse scheduling problem in these departments [6–8]. In these research studies, some soft and hard constraints are considered, including the policies of the state, rules of the hospital, and personal needs of doctors and nurses [9]. The 0-1 integer programming model, mixed integer programming model, and the goal programming model [10–13] are constructed, and the exact algorithm and heuristic algorithm are used to solve them. Musa and Saxena [14] proposed a single-stage goal programming scheduling model based on the hospital scheduling rules and the preferences of nurses. Franz and Miller [15], based on the actual situation of a large hospital, constructed a mixed integer programming model for doctor scheduling and used a bounded heuristic algorithm to get the scheduling table and put forward the adjustment strategy based on the result of the solution. Azaiez and Al Sharif [16] established a 0-1 programming model based on the constraints of the hospital regulations (nurses' skills and numbers) and the nurses' preferences (arranging night shifts and weekend shifts equally and avoiding the isolated rest days). Li et al. [17] set up a multiobjective scheduling model based on nurse rostering with many soft and hard constraints and put forward a new time-predefined metaheuristic approach called the falling tide algorithm. Bruni and Detti [18] proposed a mixed integer linear programming model that met the requirements of medical services, management rules, and personal preferences. The Branch-and-Cut procedure was used to obtain the minimization of the number of total shifts and dissatisfied shifts.

However, the problem of emergency doctor scheduling has not received much attention, and only few scholars have made preliminary exploration [19, 20]. Beaulieu et al. [21] took 6 months as a shift cycle to establish the multiobjective programming model considering the doctors' working time, the number of night shifts, and the doctors' seniority levels. The decomposition strategy can handle up to 20 doctors, but the actual scheduling table is not shown. El-Rifai et al. [22], based on the characteristics of emergency dynamic demand, set up a stochastic mixed integer programming model to achieve the best balance between the quality of service and the intensity of doctors' work. According to the fact that the patients' arrival rate is dynamic and random, Xie et al. [23, 24] used the M/M/C queuing theory to estimate the patient's waiting time in the system of dynamic demand and then constructed the mixed integer programming model for EMR scheduling to obtain a flexible shifting scheme. Besides, the data mining analysis is used to improve scheduling of EMRs [25].

To conclude, in the existing research, most of the scheduling models are based on the individual doctor or nurse as a unit, less considering the actuality of operation and management of large public hospitals in China. Besides, the depiction of the hard and soft constraints of EMR scheduling in China is insufficient. So in this paper, we consider various management rules in a hospital, physicians' personal preferences, and the time requirements of their personal learning and living to arrange their work shifts. In addition, according to the current hospital management situations in China, the doctors are divided into groups, and the multiobjective programming model is constructed to schedule physicians by groups in the emergency room. Finally, the validity of the model is validated by taking WCH as an example. The research results are expected to provide some references for scheduling EMRs in other China's high-grade large public hospitals.

This paper is organized as follows: Section 2 proposes the multiobjective scheduling model.  Section 3 gives a case study about EMRs of WCH. Results and management implications are given in Section 4. At last, the conclusions and future research directions are proposed in Section 5.

## 2. Methods

The EMRs in largest public hospitals in China are usually divided into some groups (no less than three groups). These EMRs fall into two categories: first-class doctors and second-class doctors. The work shifts include day shifts (8 a.m. to 8 p.m.), strengthen shifts (including two time intervals, 8 a.m. to 3 p.m. and 2 p.m. to 9 p.m.), and night shifts (8 p.m. on the first day to 8 a.m. on the second day). Only one doctor group works in the day shift or night shift or strengthen shift. So three doctor groups are scheduled everyday. The doctor group scheduling model is constrained by the hard constraints (national laws and hospital regulations) and soft constraints (doctors' personal preferences and flexible work rules). So taking one month as a cycle (30 days), how to schedule the doctor groups into different work shifts is a challenging problem.

In addition, considering the rationality, fairness, and humanization of EMR scheduling, some assumptions are made as follows:

- The constraints of the scheduling model are in line with labor laws in China and regulations
- Each doctor corresponds to a seniority level. There is no difference in the quality of work between doctors at the same level
- The difference of the workload of doctors at the same level in the same scheduling period is as small as possible
- The number of night shifts must be scheduled fairly and reasonably
- Doctors' personal research and teaching hours, expected rest days, and preferences for different shifts should be satisfied as much as possible

In this study, a multiobjective programming model is proposed, and the model is divided into two stages. In the first stage, the doctors are assigned to some medical teams. The doctors who want the same rest days at most are assigned into a group as much as possible. And each group must include a first-level doctor for the needs of internal exchange and learning each other within a group. In addition, the number of doctors in each group is as equal as possible. The result of grouping is obtained by the genetic algorithm. In the second stage, the assigned teams are scheduled to meet the requirements of soft and hard constraints, especially to satisfy the soft constraints as far as possible. This paper will focus on the second stage to solve the medical group scheduling problem.

### 2.1. Definition of Parameters and Variables

In order to construct the model, the parameters and variables shown in Tables 1 and 2 are applied.

### 2.2. Setting the Constraints of Formulation

According to the investigation of the ED of the largest public hospitals in China and the analysis of various factors that affect EMR scheduling, the constraints of EMR scheduling are obtained. According to whether or not the constraints must be satisfied, the constraints of EMR scheduling are divided into hard constraints and soft constraints. Hard constraints refer to the conditions that must be met in any scheduling environment; otherwise, the scheduling scheme is not feasible. Hard constraints mainly include the labor regulations and hospital management systems. Soft constraints refer to the preferences of doctors for work shifts and flexible working rules in the scheduling period.

The hard constraints of the model are as follows:

(1) $$\begin{array}{c} \overset{3}{\underset{f=1}{\sum }}x_{ijf}\leq 1,\;i=1,\ldots ,I,\;j=1,\ldots ,J, \end{array}$$

(2) $$\begin{array}{c} x_{ij3}+x_{i\left(j+1\right)1}+x_{i\left(j+1\right)2}\leq 1,\;i=1,\ldots ,I,\;j=1,\ldots ,J-1, \end{array}$$

(3) $$\begin{array}{c} \text{LK}\leq \overset{I}{\underset{i=1}{\sum }}x_{ijf}\leq \text{UK},\;j=1,\ldots ,J,\;f=1,2,3, \end{array}$$

(4) $$\begin{array}{c} \text{LD}\leq \overset{I}{\underset{i=1}{\sum }}\overset{3}{\underset{f=1}{\sum }}x_{ijf}\leq \text{UD},\;j=1,\ldots ,J, \end{array}$$

(5) $$\begin{array}{c} -A\leq \underset{j}{\sum }x_{ij3}-\underset{j}{\sum }x_{i^{\prime }j3}\leq A,\;i,\;i^{\prime }\in R_{t}, \end{array}$$

(6) $$\begin{array}{c} \underset{j\in H_{i}}{\sum }\overset{3}{\underset{f=1}{\sum }}x_{ijf}=0,\;i\in H. \end{array}$$

Hard constraints meet national laws, hospital regulations, work shifts, and working hours for doctor groups and restrictions on the number of doctor groups per shift per day. Formula (1) defines that each doctor group can only be assigned one shift in a day. Formula (2) indicates that a doctor is assigned a night shift and that no day shift or strengthen shift can be scheduled to the same doctor team the next day. Formula (3) defines that the number of doctor groups per shift should be within a certain range during the shift period. Equation (4) defines that the number of doctor groups assigned to all shifts per day should also be within a certain range in the scheduling cycle. Formula (5) defines the uniform distribution of the night shifts of doctors at the same level during the shift period. Formula (6) shows that no doctor group can be assigned any shifts during the expected rest days in the shift period. The soft constraints of the model are as follows:

(7) $$\begin{array}{c} x_{ij3}+x_{i\left(j+1\right)3}-\text{Night}1_{ij}\leq 1, \end{array}$$

(8) $$\begin{array}{c} x_{ij3}+x_{i\left(j+1\right)3}+x_{i\left(j+2\right)3}-\text{Night}2_{ij}\leq 2, \end{array}$$

(9) $$\begin{array}{c} x_{ij1}+x_{i\left(j+1\right)1}+x_{i\left(j+2\right)1}-\text{Day}1_{ij}\leq 2, \end{array}$$

(10) $$\begin{array}{c} x_{ij1}+x_{i\left(j+1\right)1}+x_{i\left(j+2\right)1}+x_{i\left(j+3\right)1}-\text{Day}2_{ij}\leq 3, \end{array}$$

(11) $$\begin{array}{c} x_{ij2}+x_{i\left(j+1\right)2}+x_{i\left(j+2\right)2}-\text{Strength}1_{ij}\leq 2, \end{array}$$

(12) $$\begin{array}{c} x_{ij2}+x_{i\left(j+1\right)2}+x_{i\left(j+2\right)2}+x_{i\left(j+3\right)2}-\text{Strength}2_{ij}\leq 3, \end{array}$$

(13) $$\begin{array}{c} x_{ij1}+x_{i\left(j+1\right)1}+x_{ij2}+x_{i\left(j+1\right)2}+x_{ij3}+x_{i\left(j+1\right)3}-2y_{ij}\leq 0,\;i=1,\ldots ,I,\;j\in S, \end{array}$$

(14) $$\begin{array}{c} x_{ij1}+x_{i\left(j+1\right)1}+x_{ij2}+x_{i\left(j+1\right)2}+x_{ij3}+x_{i\left(j+1\right)3}-y_{ij}\geq 0,\;j=1,\ldots ,I,\;j\in S, \end{array}$$

(15) $$\begin{array}{c} 4-\underset{j\in s}{\sum }y_{ij}+{\text{wkd}}_{i}\geq 1,\;i=1,\ldots ,I, \end{array}$$

(16) $$\begin{array}{c} \overset{J}{\underset{j=1}{\sum }}\overset{3}{\underset{f=1}{\sum }}x_{ijf}-pw_{i}+pt_{i}=B,\;i\in I. \end{array}$$

The soft constraints meet the flexible working rules and the doctor groups' preferences for the shifts and expected rest days. The formula (7) ensures that any one doctor group cannot be assigned two night shifts in a row during the scheduling period. If the values of the positive deviation variables Night1_*i*1_ and Night1_*i*2_ from the constraint (7) are 1, the doctor group *i* will work in three consecutive night shifts. To avoid this situation, the positive deviation variables of the constraint (8) should be given an optimum value to minimize the positive deviation variables from the constraint (7). The formulas (9) and (10) and the formulas (11) and (12) express a similar meaning to the formulas (7) and (8). The constraints (13) and (14) define that a physician group in the scheduling period has at least one full holiday weekend including Saturday and Sunday. On the basis of four weeks in the scheduling period, the value of the negative deviation variables wkd_*i*_ from the constraint (15) is also 1 in reference that the value of the binary variable *y*_*ij*_ is 1 every week. In other words, a group of doctors in the scheduling period do not have continuous rest days including Saturday and Sunday. To make any of a physician group have complete rest days on Saturday and Sunday, the deviation variables wkd_*i*_ should be minimized. The constraint (16) defines that the workload of every doctor group is evenly arranged as much as possible.

### 2.3. Building the Objective Function of the Formulation

The objective function of the model is as follows:

(17) $$\begin{array}{c} \text{Min}.\;w_{1}\overset{I}{\underset{i=1}{\sum }}\overset{J-1}{\underset{j=1}{\sum }}\text{Night}1_{ij}+w_{2}\overset{I}{\underset{i=1}{\sum }}\overset{J-2}{\underset{j=1}{\sum }}\text{Night}2_{ij}+w_{3}\overset{I}{\underset{i=1}{\sum }}\overset{J-2}{\underset{j=1}{\sum }}\text{Day}1_{ij}+w_{4}\overset{I}{\underset{i=1}{\sum }}\overset{J-3}{\underset{j=1}{\sum }}\text{Day}2_{ij}\\ w_{5}\overset{I}{\underset{i=1}{\sum }}\overset{J-2}{\underset{j=1}{\sum }}\text{Strength}1_{ij}+w_{6}\overset{I}{\underset{i=1}{\sum }}\overset{J-3}{\underset{j=1}{\sum }}\text{Strength}2_{ij}+w_{7}\overset{I}{\underset{i=1}{\sum }}{\text{wkd}}_{i}+w_{8}\overset{I}{\underset{i=1}{\sum }}Pw_{i}. \end{array}$$

The objective of this model is trying to meet most of the soft constraints and minimizing the deviation variables from the soft constraints. The deviation variables have different weight values according to the importance of the soft constraints. In this paper, each deviation variable is given a weight value which is gained by the application of the analytic hierarchy process (AHP). Suppose the value of the weight is *w*_*n*_ (*n*=1,…, 8).

## 3. Case Study

Take the No. 1 resuscitation room in the ED of WCH as a background. The composition of the doctors is shown in Table 3.

Twenty-five emergency room doctors are replaced by numbers 1∼25, of which 1∼5 and 6∼25, respectively, refer to the doctors with seniority level 1 and seniority level 2. The scheduling period is 30 days. The expected rest days of each doctor are shown in Table 4. The values of expected rest days are blank, which indicates that the corresponding doctors have no requirements of the expected rest days. Because each emergency room resident is facing many tasks simultaneously, including treating patients, scientific research, teaching, and instructing students, it is normal for different EMRs to expect different rest days.

According to the above parameters, firstly, the doctors with the same expected rest days are assigned to a group. According to the expected rest days of each doctor in Table 4, the results of grouping and arrangements of expected rest days are shown in Table 5. The number in bold indicates that the EMRs have the expected leave dates in Table 5.

## 4. Results and Management Implications

Firstly, the AHP is applied to gain the weight values of the deviation variables. Then, the CPLEX solver is used to obtain the scheduling table.

### 4.1. Application of AHP to Gain the Deviation Variable Weight Values

The criterion layer *A*_*n*_(*n*=1,…, 8) is the soft constraint of the model. The criteria are as follows: *A*_1_: no two consecutive night shifts. *A*_2_: no more than two consecutive night shifts. *A*_3_: no three consecutive day shifts. *A*_4_: no more than three consecutive day shifts. *A*_5_: no continuous three strengthen shifts. *A*_6_: no more than three continuous strengthen shifts. *A*_7_: at least one complete weekend. *A*_8_: average arrangement of each doctor's workload.

The comparisons between any two factors of the above eight factors are made by the EMRs. A total of 25 score tables are issued, and 21 of them are successfully recovered. These collected score tables include 5 copies from 5 doctors at high seniority levels (*t*=1) and 16 copies from 16 doctors at average seniority levels (*t*=2). According to the data of marking tables from 21 doctors, the weighted average formula is used to calculate the degree of importance of each constraint. Meanwhile, different weight values of doctors at the high and the average seniority level are also considered. The marking tables are only distributed to the doctors individually, and finally, the doctor group scores can be obtained through these tables.

*x*_*a*_(*a*=1,2,3,4,5) is defined as the score of importance. *x*_*a*_=1 shows that the two factors compared are equally important. *x*_*a*_=3,5,7,  *and* 9, respectively, indicates that one factor is important, strongly important, very strongly important, and extremely important than the other. On the contrary, *x*_*a*_=1/9 indicates that one factor is extremely less important than the other. *x*_*a*_=2,4,6,  *and* 8 indicates intermediary values. Suppose that the number of doctors at the seniority level *t* is *R*_*t*_. The total number of doctors at the seniority level *t* who select the importance degree *x*_*a*_ is *n*_*ta*_. The rank weight of doctors at the seniority level *t* is *w*_*t*_, *t*=1,2, ∑_*t*_ *w*_*t*_=1. The average scores $\bar{x_{t}}$ from doctors at all levels can be obtained according to the weighted average by the following equation:

(18) $$\begin{array}{c} \bar{x_{t}}=\underset{a}{\sum }\frac{x_{a}n_{ta}}{n_{t}},\;a=1,\ldots ,5,\;t=1,2. \end{array}$$

Finally, the degree of importance $\bar{x}$ of each soft constraint can be obtained by $\bar{x_{t}}$ using the following equation:

(19) $$\begin{array}{c} \bar{x}=\underset{t}{\sum }w_{t}\bar{x_{t}},\;t=1,2. \end{array}$$

Pairwise comparison of the soft constraints from 21 score tables and factors' relative importance values are shown in Table 6 (*w*_1_=0.7, *w*_2_=0.3).

According to Table 6, the judgment matrix *A* is obtained as follows:

(20) $$\begin{array}{c} A=\left[\begin{array}{cccccccc} 1 & \frac{1}{9} & \frac{1}{3} & 1 & \frac{1}{3} & 1 & \frac{1}{8} & \frac{1}{8}\\ 9 & 1 & 8 & 8 & 9 & 9 & 1 & 9\\ 3 & \frac{1}{8} & 1 & \frac{1}{9} & 1 & \frac{1}{9} & \frac{1}{8} & \frac{1}{8}\\ 1 & \frac{1}{8} & 9 & 1 & 9 & 1 & \frac{1}{7} & \frac{1}{7}\\ 3 & \frac{1}{9} & 1 & \frac{1}{9} & 1 & \frac{1}{9} & \frac{1}{8} & \frac{1}{8}\\ 1 & \frac{1}{9} & 9 & 1 & 9 & 1 & \frac{1}{7} & \frac{1}{7}\\ 8 & 1 & 8 & 7 & 8 & 7 & 1 & 8\\ 8 & \frac{1}{9} & 8 & 7 & 8 & 7 & \frac{1}{8} & 1 \end{array}\right]. \end{array}$$

The root method is applied to calculate the weight value of each criterion element as follows:

(21) $$\begin{array}{c} w={\left(0.02400,0.35280,0.02124,0.05742,0.02092,0.05658,0.32170,0.14534\right)}^{T}. \end{array}$$

The maximum eigenvalue of matrix *A* is *λ*_max_=10.5651.

The consistency index (CI) of judgment matrix *A* is found by

(22) $$\begin{array}{c} \text{CI}=\left(\frac{\lambda_{\text{max}}-n}{n-1}\right)=\left(\frac{10.5651-8}{8-1}\right)=0.3664. \end{array}$$

The random index (RI) is used as the consistency index when the matrix entries are absolutely random. Values of the random index are presented in Table 7 [26].

As the consistency evaluation index, the consistency ratio (CR) is expressed as follows:

(23) $$\begin{array}{c} \text{CR}=\frac{\text{CI}}{\text{RI}}. \end{array}$$

Referring to Table 7, when *n*=8, RI=1.41. Then, CR=0.3664/1.41=0.2598 > 0.1. So matrix *A* does not have satisfactory consistency.

After 30 corrections by the induced matrix modification method, the new judgment matrix *A*′ is as follows:

(24) $$\begin{array}{c} A^{\prime }=\left[\begin{array}{cccccccc} 1 & \frac{1}{9} & 1 & 1 & 1 & 1 & \frac{1}{8} & \frac{1}{8}\\ 9 & 1 & 8 & 8 & 9 & 9 & 1 & 9\\ 1 & \frac{1}{8} & 1 & \frac{1}{3} & 1 & \frac{1}{2} & \frac{1}{8} & \frac{1}{8}\\ 1 & \frac{1}{8} & 3 & 1 & 3 & 1 & \frac{1}{7} & \frac{1}{7}\\ 1 & \frac{1}{9} & 1 & \frac{1}{3} & 1 & \frac{1}{2} & \frac{1}{8} & \frac{1}{8}\\ 1 & \frac{1}{9} & 2 & 1 & 2 & 1 & \frac{1}{7} & \frac{1}{7}\\ 8 & 1 & 8 & 7 & 8 & 7 & 1 & 8\\ 8 & \frac{1}{9} & 8 & 7 & 8 & 7 & \frac{1}{8} & 1 \end{array}\right]. \end{array}$$

The new weight value of each criterion element is obtained as follows:

(25) $$\begin{array}{c} w^{\prime }={\left(0.03208,0.35825,0.02602,0.04430,0.02564,0.03945,0.32667,0.14759\right)}^{T}. \end{array}$$

The maximum eigenvalue of matrix *A*′ is *λ*_max_=8.9786.

(26) $$\begin{array}{c} \text{CI}=\left(\frac{8.9786-8}{8-1}\right)=0.1398. \end{array}$$

CR=0.1398/1.41=0.0991 < 0.1. So matrix *A*′ meets the requirement of satisfactory consistency. The objective function of the scheduling model can be gained, which is as follows:

(27) $$\begin{array}{c} \text{Min}.\;0.0321\overset{I}{\underset{i=1}{\sum }}\overset{J-1}{\underset{j=1}{\sum }}\text{Night}1_{ij}+0.3583\overset{I}{\underset{i=1}{\sum }}\overset{J-2}{\underset{j=1}{\sum }}\text{Night}2_{ij}+0.0261\overset{I}{\underset{i=1}{\sum }}\overset{J-2}{\underset{j=1}{\sum }}\text{Day}1_{ij}+0.0443\overset{I}{\underset{i=1}{\sum }}\overset{J-3}{\underset{j=1}{\sum }}\text{Day}2_{ij}0.0256\\ \overset{I}{\underset{i=1}{\sum }}\overset{J-2}{\underset{j=1}{\sum }}\text{Strength}1_{ij}+0.0395\overset{I}{\underset{i=1}{\sum }}\overset{J-3}{\underset{j=1}{\sum }}\text{Strength}2_{ij}+0.3267\overset{I}{\underset{i=1}{\sum }}{\text{wkd}}_{i}+0.1476\overset{I}{\underset{i=1}{\sum }}Pw_{i}. \end{array}$$

### 4.2. Using CPLEX Solver to Obtain the Scheduling Table

Considering that the mathematical model is the MILP model, the numbers of variables and constraints are small, and the data scale in this case is also small, it is appropriate to use the CPLEX solver to solve the problem. The multiobjective scheduling model was solved using IBM ILOG CPLEX 12.8 on a computer with 2.30 GHz Intel i5 processor and 64-bit 8.0 GB RAM. The solved model includes 1,310 binary variables, 10 integer variables, and 1,475 constraints. The computational time is 2.26 s.

The scheduling table is shown in Table 8. Assume that number 1 is Monday and 6 and 7, 13 and 14, 20 and 21, and 27 and 28 are weekends (represented by asterisk in Table 8). The dark shade part in Table 8 shows the expected rest days of each group. The columns BC and YB in the table show the number of total shifts and night shifts for each doctor group during the scheduling period, respectively. The doctor groups with the pentagram do not have a complete weekend. The horizontal line in Table 8 indicates that the number of consecutive shifts is more than 5 days.

Some results can be obtained by comparison of the above scheduling table with the soft constraints of the model (see Table 9). The facts of two or more consecutive night shifts and three or more consecutive day shifts and strengthen shifts do not exist in the scheduling program. Besides, the expectations of doctors for rest days are fulfilled. The soft constraint that the total shifts of the doctors at each seniority level are evenly scheduled is also generally met. Therefore, the scheduling program with the doctor group as a unit satisfies the soft and hard constraints of the model. The feasibility of the multiobjective programming model is also verified.

In the current situation in the No. 1 resuscitation room in the emergency department of West China Hospital of Sichuan University, it takes 1-2 days to construct a one-month schedule manually by trial and error. Using the proposed method in this paper, a high-quality schedule is generated in reasonable time. Besides, there may be some limitations through manual scheduling that some soft constraints cannot be satisfied. On the contrary, the scheduling result by the proposed model can be obtained in reasonable time, which can better meet various management rules in a hospital, physicians' personal preferences, and the time requirements of their personal learning and living. Therefore, the proposed model can greatly improve the efficiency and rationality of shift scheduling for China's high-grade large public hospitals.

## 5. Conclusions

Doctors are the most important medical resources in a hospital. EMRs undertake the long and intensive work. Scientific and reasonable scheduling shifts are of great significance for relieving work pressure and improving the quality of medical service. Based on the actual situation of the ED in China's high-grade large public hospitals and the fact that most of the scheduling models are based on the individual doctor as a unit and that the depiction of the hard and soft constraints of EMR scheduling in China is insufficient in the existing research, a multiobjective programming model with the doctor group as the scheduling unit is proposed aimed at satisfying the doctors' personal preferences as the soft constraint under the national laws and hospital rules. The mathematical model of the scheduling problem is to satisfy more soft constraints as far as possible. The CPLEX solver is used to obtain the scheduling table. The scheduling result satisfies the doctors' personal preferences. The feasibility and effectiveness of the method are verified by the actual case from West China Hospital of Sichuan University. The methods and ideas for scheduling EMRs can be applied to other hospitals all over the world.

Nonetheless, there are some limitations in this paper, and future research may expand further. In this paper, the AHP is applied to compute deviation variable weight values. However, the AHP is a decision-making method simulating the human brain, and it has strong subjectivity. Future research may seek more objective methods to obtain the weight values. Besides, the doctors' preferences for different shifts as the soft constraint may be considered further. Moreover, because the scheduling of the EMRs is influenced by various factors, exploring the factors that affect EMR scheduling and the index of the doctor's satisfaction further is also the direction of future research.

## Figures and Tables

**Table 1:** Notation and description of parameters.

Notation	Description
Parameters	
*I*	Set of doctor groups, indexed by *i*
*J*	Set of days of the monthly planning period, indexed by *j*
*R*	Set of doctors, indexed by *r*
*f*	Index of the shift type: *f*=1 for the day shift (8 a.m. to 8 p.m.), *f*=2 for the strengthen shift (including two time intervals, 8 a.m. to 3 p.m. and 2 p.m. to 9 p.m.), and *f*=3 for the night shift (8 p.m. on the first day to 8 a.m. on the second day)
*t*	Index of the seniority levels of the doctors: *t*=1,2 for the high and average seniority levels, respectively
*R*_*t*_	Number of doctors belonging to each seniority level: *t*=1,2
*A*	Difference in amounts of night shifts for the doctors at the same level within the planning period
*B*	Total number of shifts that a doctor group should be assigned
*H*	Set of doctor groups who need rest during the planning period
*H*_*i*_	Set of rest days of a doctor group *i*, *i* ∈ *H*
UK	Upper limit of the number of doctor groups required for every shift
LK	Lower limit of the number of doctor groups required for every shift
UD	Upper limit of the number of doctor groups required for all shifts per day
LD	Lower limit of the number of doctor groups required for all shifts per day
*S*	Set of all Saturday within the planning period

**Table 2:** Notation and description of variables.

Notation	Description
*Binary variables*	
*x*_*ijk*_	1 if the doctor group *i* is assigned to shift *k* on day *j* or 0 otherwise
*y*_*ij*_	1 if the doctor group *i* is assigned to work at least one shift on the weekend or 0 otherwise

*Positive deviation variables*	
Day1_*ij*_	1 if the doctor group *i* is assigned to the day shifts on days *j*, *j*+1, and *j*+2 consecutively or 0 otherwise
Day2_*ij*_	1 if the doctor group *i* is assigned to the day shifts on days *j*, *j*+1, *j*+2, and *j*+3 consecutively or 0 otherwise
Night1_*ij*_	1 if the doctor group *i* is assigned to the night shifts on days *j* and *j*+1 consecutively or 0 otherwise
Night2_*ij*_	1 if the doctor group *i* is assigned to the night shifts on days *j*, *j*+1, and *j*+2 consecutively or 0 otherwise
Strengthen1_*ij*_	1 if the doctor group *i* is assigned to the strengthen shifts on days *j*, *j*+1, and *j*+2 consecutively or 0 otherwise
Strengthen2_*ij*_	1 if the doctor group *i* is assigned to the strengthen shifts on days *j*, *j*+1, *j*+2, and *j*+3 consecutively or 0 otherwise

*Negative deviation variables*	
wkd_*i*_	1 if the doctor group *i* is not assigned to a complete weekend starting from Saturday within the planning period or 0 otherwise

*Positive and negative deviation variables*	
*Pw*_*i*_, *Pt*_*i*_	B shifts can be assigned to every doctor group within the planning period

**Table 3:** Parameters about doctors.

Total number of scheduled doctors	Number of doctors with seniority level 1	Number of doctors with seniority level 2
25	5	20

**Table 4:** Expected rest days of doctors.

Doctor's number	Each doctor's expected rest days' number
1, 2, 3, 4, 5	
6	15, 16, 17, 18
7, 8, 9, 10, 11	
12	2, 3, 4, 5
13	22, 23, 24
14, 15, 16	
17	10, 11, 12
18, 19, 20	
21	25, 26
22, 23, 24, 25	

**Table 5:** Results of grouping and arrangements of expected rest days.

Doctor group	Doctors	Expected rest days
A	1, 11, **12**, 18, 22	2, 3, 4, 5
B	2, 9, **13**, 16, 20	22, 23, 24
C	3, 7, 14, **17**, 24	10, 11, 12
D	4, 8, 15, **21**, 25	25, 26
E	5, **6**, 10, 19, 23	15, 16, 17, 18

**Table 6:** Pairwise comparison of the soft constraints.

	*A*_1_	*A*_2_	*A*_3_	*A*_4_	*A*_5_	*A*_6_	*A*_7_	*A*_8_
*A*_1_	1	1/9	1/3	1	1/3	1	1/8	1/8
*A*_2_	9	1	8	8	9	9	1	9
*A*_3_	3	1/8	1	1/9	1	1/9	1/8	1/8
*A*_4_	1	1/8	9	1	9	1	1/7	1/7
*A*_5_	3	1/9	1	1/9	1	1/9	1/8	1/8
*A*_6_	1	1/9	9	1	9	1	1/7	1/7
*A*_7_	8	1	8	7	8	7	1	8
*A*_8_	8	1/9	8	7	8	7	1/8	1

**Table 7:** Values of the random index (RI).

*n*	1	2	3	4	5	6	7	8	9	10
RI	0	0	0.58	0.89	1.12	1.24	1.32	1.41	1.45	1.49

**Table 8:** Scheduling table of doctor groups in the emergency room.

	Weekday					Weekend		Weekday					Weekend		Weekday					Weekend		Weekday					Weekend		Weekday		BC	YB
	1	2	3	4	5	6	7	8	9	10	11	12	13	14	15	16	17	18	19	20	21	22	23	24	25	26	27	28	29	30		
A		^*∗*^				1	3		1	2	1	3			1	3		1	3		3		2	1	1	2	1	2	3		18	6
B	1	1	3		3		2	2	3		2	1	2	3		2	3		1			^*∗*^			2	1	3			1	18	6
C	2	2	1	1	2	3			2	^*∗*^		^*∗*^			3		1	3		3		3		2	3		2	1	1	2	18	6
D	3	3		2	1	2	1	1		3		2	3	2	2	1	2	2		2	2	1	1	3	^*∗*^			3			18	6
E		3		2	1	2	1	1		3		2	3	2	^*∗*^				2	1	1	2	3			3			2	3	18	6

^*∗*^Doctor group's expected rest days.

**Table 9:** Comparison of the soft constraints with scheduling results.

Soft constraints	Results of the scheduling table
Two consecutive night shifts	0
Three consecutive night shifts	0
Three consecutive day shifts	0
Four consecutive day shifts	0
Three consecutive upper half of strengthen shifts	0
Four consecutive upper half of strengthen shifts	0
Three consecutive lower half of strengthen shifts	0
Four consecutive lower half of strengthen shifts	0
Doctors who do not have rest days at weekends	0
Doctors whose expected rest days do not meet	0
The doctor group whose total monthly work shifts are not equal to 18	0

## Data Availability

The data used to support the findings of this study are included within the article.
